# Comparison of two treatment approaches for prostate cancer: intensity‐modulated radiation therapy combined with I125 seed‐implant brachytherapy or I125 seed‐implant brachytherapy alone

**DOI:** 10.1120/jacmp.v9i2.2283

**Published:** 2008-03-18

**Authors:** Yulin Song, Maria F. Chan, Chandra Burman, Donald Cann

**Affiliations:** ^1^ Department of Medical Physics Memorial Sloan–Kettering Cancer Center Dover New Jersey U.S.A.; ^2^ Department of Radiation Oncology Memorial Sloan–Kettering Cancer Center Dover New Jersey U.S.A.

**Keywords:** Prostate cancer, brachytherapy, IMRT, PSA, IPSS

## Abstract

The purpose of the present study was to assess the results of two different treatment approaches for clinically localized prostate cancer: intensity‐modulated radiation therapy (IMRT) followed by I125 seed‐implant brachytherapy and I125 seed‐implant brachytherapy alone. We studied our 30 most recent consecutive patients. The sample population consisted of 15 cases treated with IMRT (50.4 Gy) followed by I125 seed‐implant boost (95 Gy), and 15 cases treated with I125 seed implant only (144 Gy). We analyzed established dosimetric indices and various clinical parameters. In addition, we also evaluated and compared the acute urinary morbidities of the two treatment approaches, as assessed by the international prostate symptom score (IPSS). In our series, acute urinary morbidity was slightly increased with IMRT followed by I125 seed‐implant brachytherapy as compared with I125 seed‐implant brachytherapy alone. In addition, we observed no statistically significant correlation between the IPSS and the maximum or mean urethral dose. The combination of IMRT and seed‐implant brachytherapy presents an alternative opportunity to treat clinically localized prostate cancer. The full potential of the procedure needs to be further investigated.

PACS number: 87.53.Tf

## I. INTRODUCTION

According to National Cancer Institute's 2004 progress report on prostate cancer, approximately 189 000 men were diagnosed with prostate cancer and 30 200 died from the disease in 2002.^(1)^ With increasing public awareness and widespread screening for prostate specific antigen (PSA), more patients have been diagnosed with clinically localized cancer. Although recent statistics show a stabilizing incidence and declining mortality rate, prostate cancer remains the single most common cancer in men in the United States.

Various treatment modalities exist, but no universal consensus has been reached on the best treatment for localized prostate cancer. The optimal treatment depends on the stage and histologic grade of the tumor, and the patient's age and existing medical conditions. Treatment of choice depends not only on the radiation oncologist's recommendation, but most importantly, on the patient's level of understanding of the technique and personal preference.

Currently, radical prostatectomy, androgen deprivation therapy, external‐beam radiotherapy (EBRT), and transperineal interstitial permanent ^125^I seed implantation are the major treatment options for localized prostate cancer.^(^ ^2^ ^,^ ^3^ ^)^ Although prostate brachytherapy has had a history of moderate success, new technology—particularly the introduction of ultrasound‐guided real‐time brachytherapy—has led to a revival of interest in this treatment modality and a popularity that continues to grow.

As an outpatient procedure, prostate brachytherapy has distinct advantages. It is a short and simple surgical procedure. Yet, guided by various imaging techniques, the precisely deposited radioactive seeds can create a highly conformal target dose distribution that spares the nearby organs at risk. Moreover, prostate brachytherapy is a definitive therapy with less acute morbidity and excellent biochemical disease‐free survival. Rectal complications seldom occur, and the incidence of urinary incontinence and impotence has been favorable. However, for achievement of local control, patients must have only localized disease. Generally, good candidates should have a pre‐treatment PSA below 10 ng/mL, a Gleason score (GS) below 7, a prostate volume below $60\;{cm}^{3}$, and T1–2a disease.^(4)^

Recently, rapid advances in multileaf collimator (MLC) technology and novel inverse treatment planning algorithms have created strong interest in intensity‐modulated radiation therapy (IMRT). In particular, implanted gold seed‐markers guided by an onboard kilovoltage X‐ray device offer a reliable approach to tracking prostate motion.^(2)^ The possibility for dose escalation, the optimized dose distribution, and a favorable treatment outcome have shifted the treatment of choice toward IMRT.^(3,5–8)^ Prostate cancer is now the most common tumor site treated with IMRT.

Recent studies have shown that, for low‐ or intermediate‐risk disease, radical prostatectomy, EBRT, and ^125^I transperineal interstitial seed‐implant brachytherapy are comparable with regard to long‐term biochemical disease‐free survival rates.^(^ ^9^ ^–^ ^11^ ^)^ However, for higher‐risk patients, a more aggressive treatment strategy such as a combined modality approach may be required to maximize the probability of local tumor control—and thus to improve survival.^(^ ^12^ ^,^ ^13^ ^)^

Recently, at a Memorial Sloan–Kettering Cancer Center (MSKCC) regional center, combined IMRT and ^125^I seed‐implant brachytherapy have begun to be implemented for patients with a high GS. These patients (PSA>10 ng/mL, GS>7, prostate volume $<60\;{cm}^{3}$) have a higher risk of extra‐prostatic involvement. The combination of IMRT and seed‐implant brachytherapy utilizes the advantage of both modalities. At present time, that combination is the only safe way to deliver a dose higher than 90.0 Gy—which could potentially improve treatment outcome—without severe urinary and rectal complications.

Acute urinary morbidity is the most common side effect attributable to high urethral dose. To this point, it has not been clear whether combined IMRT and seed‐implant brachytherapy could reduce acute urinary morbidity. In the present retrospective study, we evaluated and analyzed dosimetric and clinical data from our 30 most recent consecutive patients. Our objectives were to

- evaluate the IMRT and seed‐implant plans dosimetrically, using established dosimetric parameters $D_{90},D_{100}$, and $V_{100}$;
- determine the effect of urethral dose on the patients' international prostate symptom score (IPSS); and
- assess the acute urinary morbidity of combined IMRT with seed‐implant brachytherapy as compared with seed‐implant brachytherapy alone.

The study was approved by the MSKCC institutional review board.

## II. PATIENTS AND METHODS

### A. Study population

Our ^125^I transperineal interstitial seed‐implant brachytherapy program for patients with histologically confirmed adenocarcinoma of the prostate was started in 1998 at an MSKCC regional center in New Jersey. In early 2000, implementation of IMRT began in the same regional center. Between 1998 and 2004, more than 200 patients were treated with either seed implant alone, or seed implant combined with three‐dimensional conformal radiation therapy or IMRT. All cases involved clinically localized or locally advanced disease. Combined IMRT with seed‐implant treatment did not start until late 2001. For the present retrospective study, we analyzed data from our 30 most recent consecutive patients, of whom 15 received seed implants combined with IMRT and 15 received seed implants alone.

The first group of patients was treated initially with IMRT to 50.4 Gy, followed by ^125^I seed‐implant brachytherapy to an additional 95 Gy. The second group was treated with ^125^I seed‐implant brachytherapy alone, prescribed to 144 Gy. In selecting patients for each treatment approach, we used GS as the primary selection criterion and PSA as the secondary criterion. If a patient's GS was greater than 7 and his PSA was greater than 10 ng/mL, we would strongly recommend the combined modality treatment to the patient. If a patient's GS was greater than 7 and his PSA was less than 10 ng/mL, we would still recommend the combined modality treatment to the patient. If a patient's PSA was greater than 10 ng/mL and his GS was 6, we would ask the patient to consider the combined modality treatment. (This latter group of patients accounted for only a small percentage of the total study population.)

The clinical stage of the combined‐modality group ranged approximately from T1c to T2a under the American Joint Committee on Cancer staging system. In the group treated with ^125^I seed‐implant brachytherapy alone, patients had a GS below 7, a PSA below 10 ng/mL, and prostate volume below 60 cm^3^. All cases diagnosed outside MSKCC were reviewed by the institution's pathology department before simulation and treatment commenced.

### B. IMRT planning and treatment

The detailed technical aspects of simulation and of treatment planning, delivery, and quality assurance for IMRT have been described previously.^(^ ^6^ ^,^ ^7^ ^)^ Briefly, patients were simulated and scanned by computed tomography (CT) in a prone position in a customized thermoplastic mold that minimizes patient movement during the procedures. Patients were asked to drink GoLytely (Braintree Laboratories, Braintree, MA) to empty the bowel the evening before undergoing scanning. A rectal catheter was used to localize the rectum during the scanning procedure. However, a Foley catheter was not used for localization of the urethra. Computed tomography images of 3‐mm slice thickness were acquired over the pelvic region. All IMRT plans were computed using an MSKCC in‐house treatment planning system. The planning target volume (PTV), urethra, rectum, bowel, and bladder were delineated on the CT images by a radiation oncologist. The PTV was created by adding a 1‐cm margin around the clinical target volume (CTV), except at the interface of the prostate and the rectal wall, where a 6‐mm margin was used.

The planner delineated the femoral heads to include those structures in the final dose statistics. Using Boolean operations, the overlapped structures were optimized independently, so that the planner could steer hot spots away from the critical structures. Most plans consisted of 5 coplanar beams at 225 degrees, 285 degrees, 0 degrees, 75 degrees, and 135 degrees (International Electrotechnical Commission scale).

Given a set of dose limits and dose–volume constraints, plans were optimized by minimizing a quadratic objective function using an iterative gradient search algorithm.^(14)^ The quadratic objective function was constructed as the sum of squares of difference between the desired and actual doses. The algorithm computed the optimal intensity map for each beam such that the dose distribution resulting from all beams met the dose constraints specified by the planner. If the criteria for plan acceptance were not met, a trade‐off between the target dose coverage and the constraints would be made.

Once optimal intensity maps were obtained, leaf sequences were generated using the dynamic MLC (DMLC) technique.^(^ ^6^ ^,^ ^7^ ^)^ Based on the leaf sequences, the final dose distribution was then computed using a pencil‐beam algorithm.^(15)^ To minimize the urethral dose, the planner specified a dose constraint to the urethra.

Table 1 describes the criteria for plan acceptance. All patients were treated with 15‐MV photons on a Varian Clinac 21EX (Varian Medical Systems, Palo Alto, CA) equipped with a 120‐leaf MLC. Treatments were delivered in daily fractions of 1.8 Gy to a total dose of 50.4 Gy. During the course of treatment, patients were evaluated weekly by the same radiation oncologist. Acute genitourinary and gastrointestinal toxicities were scored using the Radiation Therapy Oncology Group (RTOG) morbidity grading scale.^(16)^

**Table 1:** Criteria for intensity‐modulated radiation therapy plan acceptance^a^

*Structure*		*Physical end point* (%)
PTV	$D_{\max}$	<110
	$D_{\min}$	≥87
	$D_{95}$	≥95
	$V_{95}$	≥95
Rectal wall	$D_{\max}$	≤106
	$V_{79}$	≤45
	$V_{40}$	≤90
Bladder wall	$D_{\mathrm{mean}}$	≤60
Urethra	$D_{\max}$	≤100

a These are the criteria used for 50.4 Gy prostate cases only at Memorial Sloan–Kettering Cancer Center.

PTV = planning target volume; $D_{\max}=$ maximal dose; $D_{\min}=$ minimal dose; $D_{95}=$ dose covering 95% volume; $V_{95},V_{79},V_{40}=$ volume receiving 95%,79%, and 40% prescribed dose respectively; $D_{\mathrm{mean}}=$ mean dose.

### C. ^125^I Seed‐implant brachytherapy

Following the IMRT treatments, patients again underwent CT scanning, this time in a supine position for seed‐implant planning. A balloon was inserted into the bladder and inflated with contrast agent (to approximately 10 cm^3^) to better localize the inferior border of the bladder. The urethra was easily visualized with the catheter attached to the balloon. The prostate volume was delineated on the CT images by the same radiation oncologist. The urethra and pubic bones were contoured by the planning physicist.

Based on a prescription dose of 95 Gy (144 Gy for the patient group receiving seed implant alone), the minimum numbers of needles and seeds and their coordinates were computed using an MSKCC in‐house brachytherapy planning system.^(17)^ To keep the maximum urethral dose below 165 Gy, most seeds were implanted peripherally. No seeds were permitted to be placed outside the prostate, but seeds implanted on the prostate surface were acceptable. Efforts were made to eliminate needles containing a single seed without sacrificing the target dose coverage significantly. Typically, the 95‐Gy isodose line covered the prostate with a 0.5‐cm margin, but no margin was allowed at the prostate–rectum interface. As recommended by the American Brachytherapy Society, the dosimetric parameters $V_{100}$ were computed to evaluate the quality of the plans.^(18)^ In addition, the pre‐implant maximum urethral dose, prostate volume, activity per seed, total activity, and activity per unit prostate volume were also computed.

On the day of treatment, a Mick applicator was used by the same radiation oncologist, with the participation of a urologist and the planning physicist, to implant the radioactive ^125^I seeds into the prostate under fluoroscopy guidance. During the procedure, a Foley catheter and a radio‐opaque wire were used to visualize the prostatic urethra fluoroscopically in the anterior–posterior and lateral projections. Proper needle placement with respect to the urethra was determined by comparing fluoroscopic images with projection images reconstructed from the planning CT.^(19)^

Following the implant procedure, all patients underwent post‐implant pelvic X‐ray for quality assurance purposes. The planning physicist identified the seeds on the radiographs and obtained the correct seed count. Patients then underwent CT scanning, usually 3 hours after the implant procedure, for post‐implant evaluation. The prostate and urethra were contoured by the radiation oncologist on the CT images. The post‐implant dosimetric parameters $D_{90},D_{100},V_{100}$, and the maximum and mean urethral dose and prostate volume were computed based on the CT data.

Patient follow‐up included serial PSA measurements, digital rectal examinations, and post‐treatment IPSS scores. The IPSS scores were obtained at follow‐up, 3 weeks and 4 months post implant, from reports completed by the patients. The reports were then reviewed and evaluated by the radiation oncologist. The IPSS score encodes prostate symptoms (nominal quantities) into numerical quantities that can be analyzed statistically.

### D. Data analysis and statistics

We analyzed a number of clinical parameters for acute urinary morbidity. These parameters included patient age, PSA before treatment, GS, clinical stage, prostate volume, post‐implant IPSS scores, and post‐implant PSA. In addition, we also evaluated a number of dosimetric parameters: number of needles, number of seeds, total activity, activity per unit prostate volume, $D_{90},D_{100},V_{100}$, and the maximum and mean urethral doses. For the correlation analysis between IPSS score and maximum and mean urethral doses, we used the mathematical model proposed by Singh et al.^(20)^ to convert the physical doses from the IMRT and the seed‐implant plans into a biologic effective dose (BED). For the combined‐modality group, the total dose was the sum of the IMRT BED and the seed‐implant BED. The statistical significance of the differences between the two groups was tested using the Fisher protected least‐significant difference matched‐pairs analysis of variance (StatView: SAS Institute, Cary, NC). Differences of P<0.05 were considered statistically significant.

## III. RESULTS

Table 2 compares the clinical characteristics of the two study groups. As indicated in the table, age and pre‐treatment prostate volume were similar between the two study groups (P=0.33 and P=0.68 respectively). The groups showed statistically significant differences with respect to stage and GS (P=0.018 and p<0.001 respectively). Interestingly, the groups showed no statistically significant differences in 3‐week and 4‐month IPSS score (P=0.38 and P=0.39 respectively). The mean 3‐week IPSS scores were 12.7±7.4 (combined‐modality group) and 10.2±7.1 (seed‐implant‐only group), and the mean 4‐month scores were 12.0±8.0 and 9.5±7.0. In addition, we observed no statistically significant differences between the 3‐week and 4‐month IPSS scores within each study group.

**Table 2:** Clinical characteristics of the two study groups

	*Group (each* n=15 *)*		
*Parameter*	IMRT+ *seed implant*	*Seed implant alone*	p *Value*
Age			
Mean±SD	63.0±7.1	65.7±7.6	0.33
Median	64.0	66.0	
Range	49.0∼73.0	53.0∼78.0	
Baseline PSA (ng/mL)			
Mean±SD	7.4±4.2	6.0±3.0	0.34
Median	6.92	5.1	
Range	3.3∼18.8	1.0∼14.0	
Prostate volume $\left({cm}^{2}\right)$			
Mean±SD	35.4±7.7	33.3±9.7	0.68
Median	35.8	31.7	
Range	20.8∼54.5	20.3∼52.2	
Stage	T1c∼T2a	T1c	0.018
Gleason score			
Mean±SD	6.9±0.5	6.1±0.4	<0.001
Median	7	6	
Range	6∼8	6∼7	
3‐Week IPSS			
Mean±SD	12.7±7.4	10.2±7.1	0.38
Median	15.5	7.0	
Range	2∼26	2∼25	
4‐Month IPSS			
Mean±SD	12.0±8.0	9.5±7.0	0.39
Median	13.5	8.5	
Range	2∼26	2∼22	

IMRT= intensity‐modulated radiation therapy; SD= standard deviation; PSA= prostate specific antigen; IPSS= international prostate symptom score.

**Figure 1:**
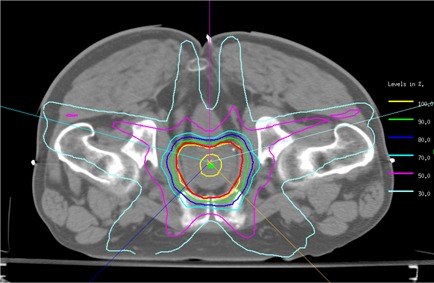
Typical isodose distribution for a representative transverse slice of an intensity‐modulated radiation therapy plan. The red and green contours represent the planning target volume (PTV) and the urethra respectively. The maximum urethral dose was 100% of the prescribed dose, thus creating a circular low‐dose region around the urethra.

Fig. 1 shows the typical isodose distribution for a representative IMRT plan. The red and green contours represent the PTV and the urethra respectively. In the illustrated plan, the 100% isodose line (yellow) covers the PTV conformably. Particularly in the region near the urethra, where a maximum dose of 100% of the prescribed dose was imposed, an excellent dose conformal avoidance was created. Applying a dose limit to the urethra was of particular importance, because the patient would subsequently be treated with seed‐implant brachytherapy.

For all IMRT plans, the mean PTV (prostate and seminal vesicles) was $35.4\pm 7.7\;{cm}^{3}$. The mean $D_{90},D_{100}$, and $V_{100}$ were 50.9±1.0 Gy, 47.5±3.0 Gy, and 93.8%±5.0% respectively. The maximum mean urethral dose was 53.8±2.0 Gy.

Table 3 compares the basic seed‐implant planning parameters for the two study groups. Significant differences were observed between the two patient groups regarding the numbers of needles and of seeds (P=0.0012 and P=0.043 respectively). That finding reflected the fact that four major factors determined the numbers of needles and seeds: prostate volume, prescription dose, seed activity, and to a lesser extent, patient anatomy.

**Table 3:** Seed implant planning parameters for the two study groups

	*Groups (each* n=15 *)*		
*Parameter*	IMRT+ *seed implant*	*Seed implant alone*	p *Value*
Needles (n)			
Mean±SD	15.2±2.0	18.4±3.2	0.0012
Median	16	18	
Range	14∼18	14∼25	
Seeds (*n*)			
Mean±SD	45.1±8.2	51.6±9.5	0.043
Median	43	52	
Range	31∼64	44∼74	
Seeds/needle			
Mean±SD	3.4±0.8	2.8±0.4	0.33
Median	2.8	2.9	
Range	2.1∼5.5	2.1∼3.4	
Activity/volume (mCi)			
Mean±SD	0.60±0.08	0.86±0.15	<0.001
Median	0.56	0.85	
Range	0.53∼0.79	0.47∼1.11	
Total activity/case (mCi)			
Mean±SD	20.93±3.75	27.49±5.5	<0.001
Median	20.45	26.88	
Range	15.62∼29.73	20.39∼40.79	

IMRT= intensity‐modulated radiation therapy; SD= standard deviation.

Given similar patient characteristics and seed activity between the two groups, the numbers of needles and seeds would be determined mainly by the prescription dose. In the present study, we used 144 Gy for the group that received seed implant alone and 95 Gy for the combined‐modality group, thus producing the significant differences. However, with respect to the number of seeds per needle, the groups showed no statistically significant difference.

We found statistically significant differences between the groups in the activity per unit prostate volume and the total activity per case (p<0.0001). These mean activities were $0.60\pm 0.08\;mCi/{cm}^{3}$ prostate volume (combined‐modality group) and $0.86\pm 0.15\;mCi/{cm}^{3}$ prostate volume (seed‐implant‐only group).

**Figure 2:**
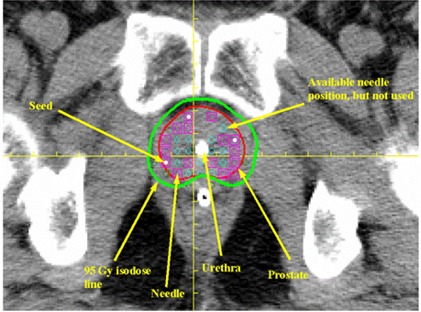
The 95‐Gy prescription isodose line (green) for a representative seed implant plan computed for the patient shown in Fig. 1. The pink squares indicate needles used for seed deposition. Three seeds were implanted in this particular slice, as indicated by three small white dots.

Fig. 2 shows the 95‐Gy prescription isodose line (green) for a representative seed‐implant plan computed for the same patient shown in Fig. 1. The red line represents the prostate and the pink squares indicate needles used for seed deposition. As shown, almost all needles were positioned peripherally. This deposition technique was employed to minimize the urethral dose. For this particular slice, three seeds were implanted as indicated by the three small white dots. Notably, a 0.5‐cm margin was created between the 95‐Gy isodose line and the prostate to account for microscopic extension of disease. However, to reduce the dose the rectum, no margin was allowed at the region anterior to that critical organ.

Table 4 shows the pre‐ and post‐implant dosimetric parameters for the combined‐modality group. The prescribed dose in the seed‐implant plans for this study group was 95 Gy. The pre‐and post‐implant prostate volumes showed no statistically significant differences, the mean values being $35.4\pm 7.7\;{cm}^{3}$ and $34.2\pm 9.9\;{cm}^{3}$ respectively. Statistically significant differences were observed between pre‐ and post‐implant values of $D_{90}$ and $D_{100}\;(p<0.001)$. In addition, pre‐ and post‐implant $V_{100}$ were statistically significantly different (p<0.001). However, the pre‐ and post‐implant maximum urethral dose showed no statistically significant differences.

**Table 4:** Basic pre‐ and post‐implant dosimetric parameters for the combined‐modality (intensity‐modulated radiation therapy plus brachytherapy seed implant) study group—prescription dose = 95 Gy

*Dosimetric parameter*	*Pre‐implant* (n=15)	*Post‐implant* (n=15)	p *Value*
Prostate volume $\left({cm}^{3}\right)$			
Mean±SD	35.4±7.7	32.7±9.8	0.38
Median	35.8	33.4	
Range	20.8∼54.5	19.3∼59.8	
$D_{90}$ (Gy)			
Mean±SD	133.7±10.2	112.2±13.4	<0.001
Median	133.5	110.5	
Range	120.9∼150.0	93.0∼145.0	
$D_{100}$ (Gy)			
Mean±SD	95.5±9.7	81.6±12.2	<0.001
Median	92.6	75.0	
Range	88.4∼120.1	75.0∼110.0	
$V_{100}$ (% prostate volume)			
Mean±SD	99.0±2.7	94.8±3.9	<0.001
Median	99.7	95.0	
Range	88.4∼73.0	88.0∼100.0	
Maximum urethral dose (Gy)			
Mean±SD	180.6±20.7	168.5±33.4	0.21
Median	174.0	160.0	
Range	152.4∼227.2	129.0∼247.4	

SD = standard deviation; $D_{90}=$ dose covering 90% volume; $D_{100}=$ dose covering 100% volume; $V_{100}=$ volume receiving 100% of the prescribed dose.

We found no correlations between $D_{90},V_{100}$, and the maximum urethral dose ($R^{2}=0.148$ and $R^{2}=0.037$ respectively). Interestingly enough, a weak correlation was seen between $D_{100}$ and the maximum urethral dose $\left(R^{2}=0.312\right)$. We also found a weak correlation between $D_{90}$ and activity per cubic centimeter of prostate volume $\left(R^{2}=0.411\right)$. Furthermore, $D_{100}$ and the maximum urethral dose correlated weakly with the number of seeds ($R^{2}=0.418$ and $R^{2}=0.367$ respectively).

Table 5 summarizes the pre‐ and post‐implant dosimetric parameters for the group that underwent seed implant alone. The prescribed dose for that group was 144 Gy. As with the combined‐modality group, we observed no statistically significant difference between the pre‐and post‐implant prostate volumes (P=0.28), with the mean values being $33.3\pm 9.7\;{cm}^{3}$ and $38.9\pm 15.1\;{cm}^{3}$ respectively. Again, we observed statistically significant differences between the pre‐ and post‐implant $D_{90},V_{100}$, and $V_{100}\;(p<0.001)$.

**Table 5:** Basic pre‐ and post‐implant dosimetric parameters for the brachytherapy‐seed‐implant‐alone study group—prescription dose = 144 Gy

*Dosimetric parameter*	*Pre‐implant* (n=15)	*Post‐implant* (n=15)	p *Value*
Prostate volume (cm^3^)			
Mean±SD	33.3±9.7	38.2±15.0	0.28
Median	31.7	38.7	
Range	20.3∼52.2	18.0∼62.8	
$D_{90}$ (Gy)			
Mean±SD	189.8±9.4	146.9±15.3	<0.001
Median	191.9	145.0	
Range	174.4∼205.1	125.0∼170.1	
$D_{100}$ (Gy)			
Mean±SD	132.1±11.8	109.2±14.5	<0.001
Median	133.1	109.0	
Range	113.0∼159.7	85.0∼136.8	
$V_{100}$ (% prostate volume)			
Mean±SD	99.7±0.4	92.8±4.7	<0.001
Median	99.7	93.0	
Range	99.4∼100.0	82.0∼98.0	
Maximum urethral dose (Gy)			
Mean±SD	243.8±24.0	224.8±38.5	0.11
Median	248.5	219.0	
Range	172.3∼279.3	158.9∼298.2	

SD = standard deviation; $D_{90}=$ dose covering 90% volume; $D_{100}=$ dose covering 100% volume; $V_{100}=$ volume receiving 100% of the prescribed dose.

The post‐implant maximum urethral dose was basically consistent with the pre‐implant value (P=0.11), the mean values being 224.8±38.0 Gy and 248.8±24.0 Gy respectively. None of the dosimetric parameters listed in Table 5 correlated significantly with the number of seeds, the number of needles, the seeds per needle, the total activity, and the activity per cubic centimeter of prostate volume, except for the maximum urethral dose, which correlated weakly with the activity per cubic centimeter of prostate volume $\left(R^{2}=0.397\right)$. Of particular importance, we found that, in both patient groups, the maximum and mean urethral doses both failed to correlate significantly with either the 3‐week or 4‐month IPSS scores, although insufficient statistical power could possibly have led to a failure to detect this difference given the sample sizes used in the study. However, the mean urethral dose was found to correlate weekly with the IPSS score for the combined‐modality group, with $R^{2}=0.40$ for the 3‐week IPSS score and $R^{2}=0.424$ for the 4‐month IPSS score. That finding could indicate that toxicity was related more to volume irradiated than to maximum dose.

Table 6 shows the results of PSA follow‐up in terms of medians and ranges for the two study groups. Following completion of the treatments, patient PSA follow‐ups were performed at 6, 12, 18, and 24 months (even longer in some cases). The median PSA for the combined modality group was 0.1 ng/mL at the 6‐month follow‐up; the corresponding value for the group receiving seed implant alone was 0.88 ng/mL. At the 12‐month follow‐up, the median PSA for the combined‐modality group was 0.04 ng/mL. The median PSA for the group receiving seed implant alone was 0.37 ng/mL. At the 18‐month follow‐up, the median PSA for both groups bounced back, being 0.16 ng/mL and 0.90 ng/mL respectively. At the 24‐month follow‐up, the mean PSA for the two groups continued to decrease.

**Table 6:** Prostate‐specific antigen (PSA) follow‐up of the two study groups

		*Groups (both* n=15 *)*			
		IMRT+ *seed implant*		*Seed implant alone*	
*Follow‐up (months)*		*Time (month)*	*PSA (ng/mL)*	*Time (month)*	*PSA (ng/mL)*
6	Median	6.0	0.10	7.1	0.88
	Range	4.3∼8.0	0.008∼0.51	5.3∼8.3	0.14∼4
12	Median	12.6	0.04	12.9	0.37
	Range	10.4∼14.7	0.026∼0.642	10.1∼14.1	0.17∼4.44
18	Median	18.4	0.16	18.0	0.90
	Range	17.4∼21.1	0.059∼0.678	16.4∼20.5	0.1∼2.6
24	Median	22.8	0.1	23.2	0.21
	Range	22.0∼24.2	0.041∼0.478	22.1∼24.2	0.1∼1.06

IMRT = intensity‐modulated radiation therapy.

## IV. DISCUSSION

Seed‐implant brachytherapy and IMRT both have distinct merits and limits. First, under image guidance, brachytherapy greatly improves seed deposition accuracy, and thus it delivers a sufficiently high dose to the macroscopic component of the target. Additionally, because of the low energy of the radioactive sources used (28 keV for I125), the dose to the surrounding normal tissues decreases very rapidly with distance and is essentially confined within a few millimeters of the prostatic capsule. Thus, seed‐implant brachytherapy provides superior normal‐tissue sparing for distant normal tissues and critical organs. However, it may simultaneously underdose any microscopic extension of the disease beyond the prostate gland. Furthermore, the target dose distribution is, in general, relatively inhomogeneous as compared with the distribution created by EBRT techniques. The phenomenon is particularly pronounced in cases in which high‐activity seeds are used and the implantation quality is not ideal. Misplacement of a high‐activity seed could result in either “hot” or “cold” spots. Prostatic cold spots and periprostatic dose deficiency can be compensated by the addition of EBRT, thus enhancing the therapeutic potential.

The IMRT technique has been known to produce highly desirable conformal dose distribution. However, accumulated dosimetric error resulting from repeated daily treatment setup uncertainty, organ movement, treatment‐induced anatomic changes, and patient weight loss during the course of treatment could compromise treatment outcome and may damage nearby normal tissues and critical organs. The risk is particularly significant in instances in which dose escalation or concurrent boost technique is employed.^(21)^ The combination of IMRT and seed‐implant brachytherapy can be dosimetrically complementary and thus could potentially improve treatment results and reduce treatment‐induced morbidity.

Relatively low morbidity is one of the reasons that patients find radiation therapy more appealing than radical prostatectomy for localized prostate cancer, given that the various available treatment options provide comparable treatment outcomes.^(22)^ Most patients tolerates EBRT or seed‐implant brachytherapy well, but as with other forms of cancer therapy, radiation‐induced side effects are still unavoidable.^(23)^ The most commonly observed side effects are acute urinary and rectal morbidity, which includes nocturia, dysuria, urinary incontinence, rectal bleeding, and diarrhea. Most patients experience the onset of acute symptoms 2 – 4 weeks into treatment. The symptoms gradually disappear 3 – 4 weeks after completion of EBRT therapy and a few months after completion of seed‐implant brachytherapy. Few patients experience long‐term symptoms or develop late complications.^(^ ^8^ ^,^ ^16^ ^)^ However, controversy exists regarding whether combined EBRT and seed‐implant brachytherapy is better than seed‐implant brachytherapy alone or whether the combined‐modality approach can yield low acute urinary and rectal morbidity.^(^ ^24^ ^,^ ^25^ ^)^

Some investigations have used combined‐modality treatment with seed‐implant brachytherapy and EBRT to attempt to maximize therapeutic gain and minimize acute urinary morbidity.^(^ ^26^ ^–^ ^28^ ^)^ Two different strategies for sequence of therapy were used: EBRT followed by seed‐implant brachytherapy as a boost^(^ ^25^ ^,^ ^26^ ^)^ and seed‐implant brachytherapy followed by EBRT as a boost.^(^ ^27^ ^,^ ^28^ ^)^ The technical limitations of the times meant that all of these studies used a static 4‐field box EBRT technique, with field sizes ranging from 8×8 cm to 12×12 cm. In some instances, custom blocks were used to block the posterior rectal wall on the lateral fields to reduce the rectal dose.^(^ ^26^ ^,^ ^28^ ^)^ The patients were treated to a total dose of either 45 Gy or 54 Gy.

The results of these studies were mixed and controversial. One study showed no statistically significant difference in early or late urinary complications between patients treated with seed‐implant brachytherapy alone and those treated with combined‐modality therapy.^(26)^ However, another study concluded that seed‐implant brachytherapy alone had fewer side effects than combined EBRT and seed‐implant treatment.^(25)^ As to rectal complications, all studies showed a higher complication rate for combined treatment, measured using the RTOG morbidity grading scale.

To date, clinical data comparing biochemical outcome and urinary symptoms between seed‐implant brachytherapy alone and combined IMRT with seed‐implant brachytherapy are scarce in the literature. Thus, the question of whether combined‐modality treatment can provide additional benefit in terms of biochemical outcome or further reduction of acute urinary morbidity (measured by IPSS score) is unclear.

In the present study, we attempted to address those issues by analyzing clinical and dosimetric parameters and their correlations with PSA and IPSS score. At the 6‐month follow‐up, median PSA for the combined‐modality group dropped from a baseline 6.92 ng/mL to 0.10 ng/mL. During the same time period, the median PSA for the group treated with seed‐implant alone decreased from a baseline 6.0 ng/mL to 0.88 ng/mL. At the 12‐month follow‐up, median PSA for the two groups continued to drop. However, the median PSA for the group treated with seed‐implant alone was higher than that for the combined‐modality group. At the 18‐ and 24‐month follow‐ups, median PSA for the combined‐modality group was slightly elevated; then, it seemed to stabilize, while median PSA for the group treated with seed‐implant alone first increased and then continued to decline further. The fast PSA response in the IMRT group could have occurred for many reasons, including temporal differences in dose deposition in the two groups and dose compensation by IMRT.

It has been suggested that urinary symptoms following radiation therapy might be related to the maximum dose delivered to the urethra.^(29)^ However, several recent studies indicated a lack of correlation between the maximum urethral dose and urinary symptoms or IPSS score.^(^ ^30^ ^–^ ^32^ ^)^

Bucci et al.^(30)^ reported that, in a multivariate analysis, no dosimetric parameters were correlated with the requirement for post‐implantation catheterization in patients having obstructive urinary symptoms. Those authors concluded that only baseline IPSS score was the most significant predicative factor for post‐implantation catheterization.

In a retrospective study, Crook et al.^(31)^ analyzed the results of 150 consecutive patients treated with ^125^I seed‐implant brachytherapy. Among those 150 patients, 20 (13%) experienced acute urinary retention (AUR). The authors found that none of the dosimetric parameters, including $D_{90},V_{100},V_{200}$, and maximum urethral dose, was predictive of AUR. In addition, they found that baseline IPSS score did not correlate with AUR after implantation. Prostate volume was the major determinant of AUR.

A report on 172 patients by Cesaretti et al.^(32)^ showed a mean pre‐treatment IPSS score of 7.5 and a mean peak IPSS score post‐implantation of 19.4. As determined by IPSS score, 35.5% of the patients experienced a urinary symptom. The study found that no single clinical or implant parameter, including PSA, disease stage, use of hormone therapy, seed activity, prostate volume, seed number, or urethral dose, was statistically significantly correlated with urinary symptoms or IPSS score.

In a prospective randomized trial that combined EBRT with seed‐implant brachytherapy, Merrick et al.^(33)^ found that the isotope type, supplemental EBRT, and maximum urethral dose did not significantly correlate with post‐treatment IPSS score and thus did not predict dysuria.

In our current series, the data also showed that no clinical or dosimetric parameter was significantly correlated with IPSS score for either the combined‐modality group or the group treated with seed‐implant alone. Those results agree with the findings of the earlier investigators. Nevertheless, our data showed a weak positive linear correlation between IPSS score and total maximum urethral dose. In addition, our data also revealed, in the combined‐modality group, a week linear correlation between IPSS score and mean urethral dose. All of those findings suggest that a reduction in mean or maximum urethral dose may result in fewer urinary symptoms and that combined‐modality treatment could induce a higher rate of acute urinary morbidity. That result seems to be consistent with the popular assumption that combined EBRT and seed‐implant brachytherapy results in a higher rate of urinary complications than does either therapy alone.^(23)^

We must point out that post‐implant dosimetry in the present study was performed 3 hours after the implant procedure, rather than 1 month after, as has been widely adopted. This timing was chosen for the sake of patient convenience. We are aware of the potential effects of post‐implant edema and possible seed migration on the accuracy of post‐implant dosimetry. Based on the published studies^(^ ^34^ ^,^ ^35^ ^)^ and our own experience,^(^ ^36^ ^,^ ^37^ ^)^ we believe that our post‐implant dosimetry data provide a very conservative estimation of several important dosimetric parameters, such as $D_{90},D_{100}$, and $V_{100}$. For that reason (among others), our post‐implant dosimetry was not favorable as compared with the pre‐implant dosimetry.

It has been suggested that the dose from above the basal prostate level to the bladder base (bladder neck), rather than the dose to the urethra, could be the stronger predictor of acute urinary morbidity^(38)^—being that this area of the bladder is sensitive to radiation and is often covered by high isodose lines. Thus, it may be beneficial to restrict the dose to that area as much as possible.

## V. CONCLUSIONS

As a curative therapy, brachytherapy has a long history of being used to treat localized prostate cancer. Early results seemed to be disappointing because of immature technology and inappropriate patient selection. Rapid advancements in the technology of medical imaging and treatment planning systems and a better understanding of prognostic factors have revitalized the technique. As a result, brachytherapy is emerging as an indispensable treatment option. Combined IMRT with seed‐implant brachytherapy is particularly becoming very appealing to patients who are not willing to undergo radical prostatectomy. Combined therapy has been shown to be the safest way to deliver a dose higher than 90.0 Gy. However, successful implementation of the technique requires extensive expertise from several different disciplines, a situation that may make such implementation infeasible for centers with limited medical physics support.

Our preliminary study showed that IMRT followed by ^125^I seed‐implant brachytherapy is a promising and viable treatment technique for patients with high‐grade and clinically localized prostate cancer. However, as compared with ^125^I seed‐implant brachytherapy alone, it could increase acute urinary morbidity as assessed by IPSS score. We observed no statistically significant correlation between IPSS score and the mean and maximum urethral doses. However, we must point out that, because the sample sizes in this study were relatively small, it was difficult, in a statistical sense, to accurately determine the effects of urethral dose on IPSS score and to make a definitive conclusion regarding treatment outcomes. Thus, further studies using larger sample sizes are needed to validate these preliminary findings. Nevertheless, we hope that the results presented here could function as a useful reference for others. We believe that IMRT combined with seed‐implant brachytherapy presents an alternative opportunity to treat prostate cancer. It utilizes the advantages of both modalities. Its full potential needs to be further investigated.

## Supporting information

Supplementary Material

Supplementary Material

Supplementary Material
