# Supplementary figures and images for: Comparison of two treatment approaches for prostate cancer: intensity‐modulated radiation therapy combined with I125 seed‐implant brachytherapy or I125 seed‐implant brachytherapy alone
Source: J Appl Clin Med Phys. 2008 Mar 18;9(2):1–14. doi: 10.1120/jacmp.v9i2.2283 (PMC5721712; doi:10.1120/jacmp.v9i2.2283)

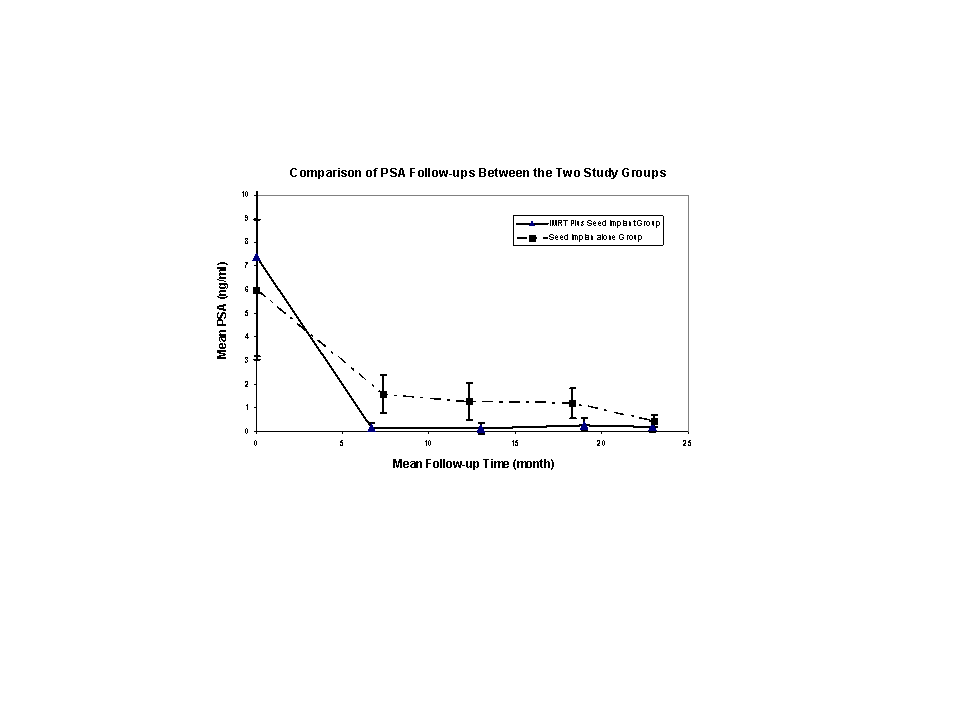

Supplement: Supplementary file 1 — Supplementary Material [file ACM2-9-001-s001.gif]
