# Supplementary material for: Comparison of two treatment approaches for prostate cancer: intensity‐modulated radiation therapy combined with I125 seed‐implant brachytherapy or I125 seed‐implant brachytherapy alone
Source: J Appl Clin Med Phys. 2008 Mar 18;9(2):1–14. doi: 10.1120/jacmp.v9i2.2283 (PMC5721712; doi:10.1120/jacmp.v9i2.2283)

Comparison of Two Treatment Approaches for

Prostate Cancer: Combined Intensity Modulated Radiation Therapy with ^125^I Seed Implant Brachytherapy and ^125^I Seed Implant Brachytherapy alone

**Yulin Song, Ph.D.^1^*, Maria F. Chan, Ph.D.^1^,**

**Chandra Burman, Ph.D.^1^, and Donald Cann, MD.^2^**

*^1^Department of Medical Physics*

*Memorial Sloan-Kettering Cancer Center at Dover, NJ*

*400 Blackwell Street, NJ 07801*

*^2^Department of Radiation Oncology*

*Memorial Sloan-Kettering Cancer Center at Dover, NJ*

*400 Blackwell Street, NJ 07801*

[songy@mskcc.org](mailto:songy@mskcc.org), [chanm@mskcc.org](mailto:chanm@mskcc.org), [burmanc@mskcc.org](mailto:burmanc@mskcc.org), [dcann@saintclares.org](mailto:dcann@saintclares.org)

Running Title: Combined IMRT with Brachytherapy for Prostate Cancer

***Address Correspondence to:**

Yulin Song, Ph.D.

Department of Medical Physics

Memorial Sloan-Kettering Cancer Center at Phelps

777 N Broadway, Suite 100

Sleepy Hollow, NY 10591

Tel: (914)-333-8676

Fax: (914)-887-8506

E-mail: songy@mskcc.org

Comparison of Two Treatment Approaches for Prostate Cancer: Combined Intensity Modulated Radiation Therapy with ^125^I Seed Implant Brachytherapy and ^125^I Seed Implant Brachytherapy alone

**Abstract:** The purpose of this study was to assess the results of two different treatment approaches for clinically localized prostate cancer: intensity modulated radiation therapy (IMRT) followed by ^125^Iodine (^125^I) seed implant brachytherapy and ^125^I seed implant brachytherapy alone. Thirty most recent consecutive patients were studied. The sample population consisted of 15 cases treated with IMRT (50.4 Gy), followed by ^125^I seed implant boost (95 Gy), and 15 cases treated with ^125^I seed implant only (144 Gy). Established dosimetric indices and various clinical parameters were analyzed. In addition, the acute urinary morbidities of the two treatment approaches, as assessed by the International Prostate Symptom Score (IPSS), were also evaluated and compared. In our series, IMRT followed by ^125^I seed implant brachytherapy slightly increased the acute urinary morbidity compared with ^125^I seed implant brachytherapy alone. In addition, there was no statistically significant correlation between the IPSS and the maximum or mean urethral dose. Combination of IMRT and seed implant brachytherapy presents an alternative opportunity to treat clinically localized prostate cancer. Its full potential needs to be further investigated.

Keywords: Prostate cancer, brachytherapy, IMRT, PSA, and IPSS

PACS: 87.53.Tf

© 2007 American College of Medical Physics

**Introduction**

According to National Cancer Institute’s (NCI) 2004 progress report on prostate cancer, approximately 189,000 men were diagnosed with prostate cancer and 30,200 died from this disease in 2002^(1)^. With increasing public awareness and widespread prostate specific antigen (PSA) screening, more patients have been diagnosed with clinically localized cancer. While recent statistics show stabilizing incidence and declining mortality rates, prostate cancer still remains the single most common cancer in men in the United States. Though various different treatment modalities exist, there is no universal consensus on the best treatment for localized prostate cancer. The optimal treatment depends on the stage and histological grade of the tumor, the patient age, and existing medical conditions. The final treatment of choice depends not only on the radiation oncologist’s recommendation, but, most importantly, on the patient’s level of understanding of the technique and personal preference.

Currently, radical prostatectomy, androgen deprivation therapy, external beam radiotherapy (EBRT), and transperineal interstitial permanent ^125^I seed implantation are the major treatment options for localized prostate cancer^(2, 3)^. Although prostate brachytherapy has a history of moderate success, with new technology and particularly, the introduction of ultrasound-guided real-time brachytherapy, interest in this treatment modality has regained popularity and continues to grow. As an outpatient procedure, prostate brachytherapy has its distinct advantages. It is a short and simple surgical procedure. Yet, guided by various imaging techniques, the precisely deposited radioactive seeds can create a highly conformal target dose distribution while sparing the nearby organs at risk (OAR). It is a definitive therapy with less acute morbidity and excellent biochemical disease-free survival. Rectal complications have seldom occurred. Furthermore, incidence of urinary incontinence and impotence has been favorable. However, to achieve local control, patients must only have localized disease. Generally, good candidates should have pre-treatment PSA < 10 ng/ml, Gleason score < 7, prostate volume < 60 cm^3^, and T1-2a disease^4^. Recently, rapid advances in multileaf collimator (MLC) technology and novel inverse treatment planning algorithms have created a strong interest in intensity modulated radiation therapy (IMRT). In particular, implanted gold seed markers guided by the on-board kV x-ray offer a reliable approach to track the prostate motion ^(2)^. The possible dose escalation, optimized dose distribution, and favorable treatment outcome have shifted the treatment of choice toward IMRT ^(3, 6-8)^. Now, prostate cancer is the most common tumor site treated with IMRT.

Recent studies have shown that for low or intermediate risk disease, radical prostatectomy, EBRT, and ^125^Iodine (^125^I) transperineal interstitial seed implant brachytherapy are comparable in long-term biochemical disease-free survival rates ^(9-11)^. For higher risk patients, however, a more aggressive treatment strategy, such as combined modality approach, may be required to maximize the probability of local tumor control and thus, improve patients’ survival ^(12, 13)^. Recently, at a Memorial Sloan-Kettering Cancer Center (MSKCC) regional center, we started implementing combined IMRT and ^125^I seed implant brachytherapy for patients with high Gleason score. These patients (PSA > 10 ng/ml, Gleason score (GS) > 7, prostate volume < 60 cm^3^) had a higher risk of extra-prostatic involvement. By combining IMRT and seed implant brachytherapy, we took advantages of both modalities. It is, at present time, the only safe way to deliver a dose higher than 90.0 Gy without severe urinary and rectal complications, which could potentially improve treatment outcome.

Acute urinary morbidity is the most common side effect that could be attributed to high urethral dose. Up to now, it is not clear whether combined IMRT and seed implant brachytherapy could potentially reduce the acute urinary morbidity. In this retrospective study, dosimetric and clinical data from 30 most recent consecutive patients were evaluated and analyzed. The objectives of this study were 1) to evaluate the IMRT and seed implant plans dosimetrically using established dosimetric parameters D_90_, D_100_, and V_100_ and to determine the effect of urethral dose on the International Prostate Symptom Score (IPSS), 2) to assess the acute urinary morbidities of combined IMRT with seed implant brachytherapy and seed implant brachytherapy alone. The study has been approved by the MSKCC Institutional Review Board (IRB)

**Materials and Methods**

***Patients***

In 1998, we started our ^125^I transperineal interstitial seed implant brachytherapy program for patients with histologically confirmed adenocarcinoma of the prostate at an MSKCC regional center in New Jersey. In early 2000, we began implementing IMRT in this regional center. Between 1998 and 2004, more than 200 patients have been treated with either seed implant alone or seed implant combined with 3D conformal radiation therapy (3-DCRT) or IMRT. All cases were clinically localized or locally advanced diseases. Combined IMRT with seed implant treatment did not start until late 2001. Data of thirty most recent consecutive patients were analyzed for this retrospective study. Fifteen were seed implant combined with IMRT and 15 were seed implant alone. The first group of patients was initially treated with IMRT to 50.4 Gy, followed by ^125^I seed implant brachytherapy to additional 95 Gy. The second group was treated with ^125^I seed implant brachytherapy alone, prescribed to 144 Gy. In selecting patients for each treatment approach, we used GS as the primary selection criterion and PSA as the secondary selection criterion. This means that if a patient’s GS was greater than 7 and PSA was greater than 10 ng/ml, we would strongly recommend the patient for the combined modality treatment. If a patient’s GS was greater than 7 and PSA was less than 10 ng/ml, we would still recommend the patient for the combined modality treatment. On the other hand, if a patient’s PSA was greater than 10 ng/ml and his GS was 6, we would also ask the patient to consider the combined modality treatment, but this group of patients only accounted for a small percentage of the total studied population. The clinical stage of the combined modality group ranged from T1c ~ T2a using the American Joint Committee on Cancer (AJCC) staging system. For the ^125^I seed implant brachytherapy alone group, patients had GS < 7, PSA < 10 ng/ml, and prostate volume < 60 cm^3^. All the cases diagnosed outside MSKCC were reviewed by our Pathology Department prior to commencing simulation and treatment.

***IMRT Planning and Treatment***

The detailed technical aspects for the simulation, treatment planing, delivery, and quality assurance for the IMRT have been described previously ^(6, 7)^. Briefly, patients were simulated and CT scanned in a prone position in a customized thermoplastic mold to minimize patient movement during the procedures. Patients were asked to drink Golytely to empty the bowel the evening before the CT scanning. A rectal catheter was used to localize the rectum during the CT scanning. However, the Foley catheter was not used for the localization of the urethra. CT images of 3 mm slice thickness were acquired over the pelvic region. All IMRT plans were computed using an MSKCC in-house treatment planning system. The planning target volume (PTV), urethra, rectum, bowels, and bladder were delineated on the CT images by a radiation oncologist. The PTV was created by adding 1 cm margin around the clinical target volume (CTV), except at the prostate and rectal wall interface where a 6 mm margin was used.

The planner delineated the femoral heads to include the structures in the final dose statistics. By Boolean operations, the overlapped structures were optimized independently, which allowed the planner to steer hot spots away from the critical structures. Most plans consisted of five co-planner beams, 225°, 285°, 0°, 75° and 135° in IEC scale. Given a set of dose limits and dose-volume constraints, plans were optimized by minimizing a quadratic objective function using an iterative gradient search algorithm ^(14)^. The quadratic objective function was constructed as the sum of squares of difference between the desired and actual doses. The algorithm computed the optimal intensity map of each beam such that the resultant dose distribution from all beams met the dose constraints specified by the planner. If the criteria for plan acceptance were not met, a trade-off between the target dose coverage and constraints would have to be made. Once optimal intensity maps were obtained, leaf sequences were generated using the dynamic MLC (DMLC) technique ^(6, 7)^. Based on these leaf sequences, the final dose distribution was then computed using a pencil beam algorithm ^(15)^. To minimize the urethral dose, the planner specified a dose constraint to the urethra.

Criteria for plan acceptance are described in Table 1. All patients were treated with 15 MV photons on a Varian Clinac 21EX (Palo Alto, CA) equipped with a 120-leaf MLC. Treatments were delivered in daily fractions of 1.8 Gy to a total dose of 50.4 Gy. During the course of treatment, patients were evaluated by the same radiation oncologist weekly. Acute genitourinary (GU) and gastrointestinal (GI) toxicities were scored using the Radiation Therapy Oncology Group (RTOG) morbidity grading scale ^(16)^.

***^125^I Seed Implant Brachytherapy***

Following the IMRT treatments, the patients were CT scanned again in a supine position for seed implant planning. A balloon was inserted into the bladder and then inflated with ~ 10 cm^3^ contrast agent to better localize the inferior border of the bladder. The urethra was easily visualized with the catheter attached to the balloon. The prostate volume was delineated on the CT images by the same radiation oncologist. The urethra and pubic bones were contoured by the planning physicist. Based on a prescription dose of 95 Gy, the minimum numbers of needles and seeds and their coordinates were computed using an MSKCC in-house brachytherapy planning system ^(17)^. To keep the maximum urethral dose below 165 Gy, most seeds were implanted peripherally. No seeds were placed outside the prostate, but seeds implanted on the prostate surface were acceptable. Efforts were made to eliminate needles containing a single seed without sacrificing the target dose coverage significantly. Typically, the 95 Gy isodose line covered prostate with a 0.5 cm margin, but no margin was allowed at the prostate-rectum interface. The dosimetric parameters D_90_, D_100_, and V_100_, as recommended by the American Brachytherapy Society (ABS), were computed to evaluate the quality of the plans ^(18)^. In addition, the pre-implant maximum urethral dose, prostate volume, activity per seed, total activity, and activity per unit prostate volume were also computed. For seed implant alone patient group, the prescribed dose was 144 Gy.

On the day of the treatment, radioactive ^125^I seeds were implanted into the prostate with a Mick Applicator under fluoroscopy guidance by the same radiation oncologist, with the participation of a urologist and the planning physicist. During the procedure, a Foley catheter, along with a radio-opaque wire, was used to visualize the prostatic urethra fluoroscopically in anterior-posterior and lateral projections. Proper needle placement was determined by comparing fluoroscopic images to projection images reconstructed from planing CT with respect to the urethra ^(19)^. Following the implant, all patients had their post-implant pelvis x-ray for quality assurance purpose. The planning physicist identified the seeds on the x-ray films and obtained the correct seed count. Patients were then CT scanned, usually 3 hours following the implant procedure for post-implant evaluation. The prostate and urethra were contoured by the radiation oncologist on the CT images. The post-implant dosimetric parameters D_90_, D_100_, V_100_, the maximum and mean urethral dose, and prostate volume were computed based on the CT data. Patient follow-up included serial PSA measurements and digital rectal examinations (DRE), and post-treatment IPSS scores. The IPSS scores were obtained at follow-ups at 3 weeks and 4 months post implant from reports filled by the patients and were then reviewed and evaluated by the radiation oncologist. IPSS score encodes the prostate symptoms, the nominal quantities, into numerical quantities that can be analyzed statistically.

***Data Analysis and Statistics***

A number of clinical parameters were analyzed for acute urinary morbidity. These included patient age, pre-treatment PSA, GS, clinical stage, prostate volume, and post-implant IPSS scores and PSA. In addition, a number of dosimetric parameters were also evaluated, which consisted of number of needles, number of seeds, total activity, activity per unit prostate volume, D_90_, D_100_, V_100_, and the maximum and mean urethral dose. For the correlation analysis between IPSS and the maximum and mean urethral doses, the physical dose of both IMRT and seed implant plans were converted into biological effective dose (BED) using the mathematical model proposed by Singh *et al*. ^(20)^. For the combined modality treatment group, the total dose was the sum of IMRT BED and seed implant BED. The statistical significance of the differences between the two groups was tested using Fisher’s PLSD Matched-pairs analysis of variance (ANOVA) (Statview, SAS Inst.). Differences of *p* < 0.05 were considered statistically significant.

**Results**

The comparison of clinical characteristics between the two study groups is shown in Table 2. As indicated in the table, the age and the pre-treatment prostate volume were similar between the two study groups with *p* = 0.33 and 0.68, respectively. There were statistically significant differences between the two study groups with respect to stage and GS with *p* = 0.018 and < 0.001, respectively. Interestingly, there were no statistically significant differences in 3-week and 4-month IPSSs between the two study groups (*p* = 0.38 and 0.39, respectively). The mean 3-week IPSSs were 12.7±7.4 and 10.2±7.1 for the combined modality group and the seed implant alone group, respectively. The mean 4-month IPSSs were 12.0±8.0 and 9.5±7.0 for the combined modality group and the seed implant alone group, respectively. In addition, there were no statistically significant differences between 3-week and 4-month IPSS values within each study group. Figure 1 shows the typical isodose distribution for a representative IMRT plan. The red and green contours represent the PTV and the urethra, respectively. In this plan, the 100% isodose line (yellow) covered the PTV conformably. Particularly, in the region near the urethra where a maximum dose of 100% of the prescribed dose was imposed, an excellent dose conformal avoidance was created. Applying a dose limit to the urethra was of particular importance because the patient would be treated subsequently by the seed implant brachytherapy. For all IMRT plans, the mean PTV (prostate + seminal vesicles) was 35.4±7.7 cm^3^. The mean D_90_, D_100_, and V_100_ were 50.9±1.0 Gy, 47.5±3.0 Gy, and 93.8±5.0%, respectively. The mean maximum urethral dose was 53.8±2.0 Gy.

Table 3 compares the basic seed implant planning parameters for the two study groups. Significant differences were observed between the two patient groups regarding the number of needles and the number of seeds (*p* = 0.0012 and 0.043, respectively). This was due to the fact that four major factors determined the number of needles and number of seeds: the prostate volume, the prescription dose, the seed activity, and, to a lesser extent, the patient anatomy. Given the similar patient characteristics and seed activity between the two groups, the number of needles and number of seeds would be mainly determined by the prescription dose. In our study, we used 144 Gy for the seed implant alone group and 95 Gy for the combined modality group, thus resulting in these significant differences. However, there was no statistically significant difference between the two patient groups with respect to the number of seeds per needle. It was found that there were statistically significant differences in the activity per unit prostate volume and the total activity per case (*p* <0.0001). The mean activities per unit prostate volume were 0.60±0.08 mCi/cm^3^ and 0.86±0.15 mCi/cm^3^ for the combined modality group and the seed implant alone group, respectively. Figure 2 shows the 95 Gy prescription isodose line (green) for a representative seed implant plan computed for the same patient as shown in Figure 1. The red line represents the prostate and the pink squares indicate needles used for seed deposition. As shown, almost all needles were positioned peripherally. This deposition technique was employed to minimize the urethral dose. For this particular slice, three seeds were implanted, as indicated by three small white dots. Of particular note, a 0.5 cm margin was created between the 95 Gy isodose line and the prostate to account for the microscopic extension of the disease. However, no margin was allowed at the region anterior to the rectum to reduce the dose to this critical organ.

Table 4 shows the pre- and post-implant dosimetric parameters for the combined modality group. The prescribed dose for the seed implant plans for this study group was 95 Gy. There was no statistically significant difference between the pre- and post-implant prostate volumes, with the mean being 35.4±7.7 cm^3^ and 34.2±9.9 cm^3^, respectively. Statistically significant differences were observed between pre- and post-implant D_90_ and D_100_ (*p* < 0.001). In addition, there was a statistically significant difference regarding pre- and post-implant V_100_ (*p* < 0.001). However, there was no statistically significant difference between pre- and post-implant maximum urethral dose. It was found that there were no correlations between D_90_, V_100_, and the maximum urethral dose (*R*^2^ = 0.148 and 0.037, respectively). Interestingly enough, there was a weak correlation between D_100_ and the maximum urethral dose (*R*^2^ = 0.312). It was also found that there was a weak correlation between D_90_ and activity per cubic centimeter prostate volume (*R*^2^ = 0.411). Furthermore, D_100_ and the maximum urethral dose correlated weakly with the number of seeds (*R*^2^ = 0.418 and 0.367, respectively).

Table 5 summarizes the pre- and post-implant dosimetric parameters for the seed implant alone group. For this study group, the prescribed dose was 144 Gy. Like the combined modality treatment group, there was no statistically significant difference between the pre- and post-implant prostate volumes (*p* = 0.28), with the mean being 33.3±9.7 cm^3^ and 38.9±15.1 cm^3^, respectively. Again, we observed statistically significant differences between pre- and post-implant D_90_, D_100_, and V_100_ (*p* < 0.001). The post-implant maximum urethral dose was basically consistent with the pre-implant value (*p* = 0.11), with the mean values being 224.8±38.0 Gy and 243.8±24.0 Gy, respectively. None of the dosimetric parameters listed in Table 5 correlated significantly with the number of seeds, the number of needles, the seeds per needle, the total activity, and the activity per cubic centimeter prostate volume except for the maximum urethral dose, which correlated weakly with the activity per cubic centimeter prostate volume (*R*^2^ = 0.397). Of particular importance, it was found that both maximum and mean urethral doses did not correlate significantly with either 3-week or 4-month IPSS for both patient groups, although there was a possibility that insufficient statistical power could fail to detect this difference, given the sample sizes used in this study. However, the mean urethral dose was found to correlate weekly with IPSS for the combined modality group, with R^2^ = 0.40 for the 3-week IPSS and R^2^ = 0.424 for the 4-month IPSS. This could indicate that the toxicity was affected more by volume irradiated than by the maximum dose.

Table 6 shows the results of PSA follow-up for the two study groups in terms of medians and ranges. Following completion of the treatments, the patient PSA follow-ups were performed at 6, 12, 18, and 24 months or even a longer time in some cases. The median PSA for the combined modality group was 0.1 ng/ml at the 6-month follow-up, while the corresponding value for the seed implant alone group was 0.88 ng/ml. At the 12-month follow-up, the median PSA for the combined modality group was 0.04 ng/ml. The median PSA for the seed implant alone group was 0.37 ng/ml. At the 18-month follow-ups, the median PSA for both groups bounced back, being 0.16 and 0.90 ng/ml, respectively. At the 24-month follow-ups, the mean PSA for the two groups continued decreasing.

**Discussion**

Both seed implant brachytherapy and IMRT have its distinct merits and limits. First of all, under the image guidance, brachytherapy greatly improves seed deposition accuracy and thus, deliver sufficiently high dose to the macroscopic component of the target. Additionally, due to the low energy of these radioactive sources (28 keV for ^125^I), the doses to the surrounding normal tissues decrease very rapidly with distance and are essentially confined within a few millimeters of the prostatic capsule. Thus, it provides a superior normal tissue sparing for distant normal tissues and critical organs. However, it may, at the same time, underdose the microscopic extension of the disease beyond the prostate gland. Furthermore, the target dose distribution is, in general, relatively inhomogeneous compared with that created by EBRT techniques. This phenomenon is particularly pronounced in cases where high activity seeds are used and the implantation quality is not ideal. Misplacement of a high activity seed could result in either hot spots or cold spots. Nevertheless, these prostatic cold spots and periprostatic dose deficiency can be compensated for by the addition of EBRT, thus, enhancing the therapeutic potential. IMRT has been known to produce highly desirable conformal dose distribution. However, accumulated dosimetric error resulting from repeated daily treatment set-up uncertainty, organ movement, treatment-induced anatomic changes, and patient weight loss during the course of treatment could compromise the treatment outcome and may damage the nearby normal tissues and critical organs. This risk is particularly significant in instances where the dose escalation or concurrent boost techniques are employed ^(21)^. The combination of IMRT and seed implant brachytherapy can complement each other dosimetrically and thus, could potentially improve treatment result and reduce treatment-induced morbidity.

Relatively low morbidity is one of the appealing reasons why patients choose radiation therapy over radical prostatectomy for localized prostate cancer when various available treatment options give comparable treatment outcomes ^(22)^. Majority of patients tolerates EBRT or seed implant brachytherapy well, but radiation-induced side effects are still unavoidable as in the case with other forms of cancer therapy ^(23)^. The most commonly observed side effects are acute urinary and rectal morbidity, which includes nocturia, dysuria, urinary incontinence, rectal bleeding, and diarrhea. Most patients feel the onset of acute symptoms 2-4 weeks into the treatment. The symptoms gradually disappear 3-4 weeks for EBRT and a few months for seed implant brachytherapy following completion of the therapy. Few patients experience long-term symptoms or develop late complications ^(8, 16)^. However, controversy exists regarding whether combined EBRT and seed implant brachytherapy is better than seed implant brachytherapy alone or whether combined modality approach can yield low acute urinary and rectal morbidity ^(24, 25)^. There have been some investigations of combined modality treatment utilizing seed implant brachytherapy and EBRT to attempt to maximize the therapeutic gain and minimize the acute urinary morbidity ^(26-28)^. In terms of the sequence of the therapy, there were two different strategies; EBRT followed by seed implant brachytherapy as a boost ^(25, 26)^ or seed implant brachytherapy followed by EBRT as a boost ^(27, 28)^. Due to technical limitation of the time, all these studies used a static 4-field box technique as EBRT, with field sizes ranging from 8×8 cm to 12×12 cm. In some instances, custom blocks were used to block the posterior rectal wall on the lateral fields to reduce the rectal dose ^(26, 28)^. The patients were treated to a total dose of either 45 Gy or 54 Gy. The results of these studies were mixed and controversial. One study showed no statistically significant difference in early or late urinary complications between seed implant brachytherapy alone and combined modality treated patients ^(26)^. However, another study concluded that seed implant brachytherapy alone had fewer side effects than combined EBRT and seed implant treatment ^(25)^. As to the rectal complications, all studies showed a higher complication rate for combined treatment, measured by RTOG morbidity grading scale.

Up to now, there is a scarcity of clinical data in the literature comparing the biochemical outcome and urinary symptoms between seed implant brachytherapy alone and combined IMRT with seed implant brachytherapy. Thus, it is unclear whether this combined modality treatment can provide additional treatment benefit in terms of biochemical outcome or it can further reduce acute urinary morbidity in terms of IPSS. In our current study, we attempted to address these issues by analyzing the clinical and dosimetric parameters and their correlations with PSA and IPSS. At the 6-month follow-up, the median PSA for the combined modality group dropped from baseline 6.92 ng/ml to 0.10 ng/ml. During the same time period, the median PSA for the seed implant alone group decreased from baseline 6.0 ng/ml to 0.88 ng/ml. At the 12-month follow-up, the median PSAs for the two groups continued to drop. However, the median PSA for the seed implant alone group was higher than that for the combined modality group. At the 18- and 24-month follow-ups, the median PSAs for the combined modality group were slightly elevated and, then, seemed to stabilize, while the median PSAs for the seed implant alone group increased first and then continued to decrease further. The fast PSA response in the IMRT group could be due to many reasons. These include the temporal differences in dose deposition in the two groups and dose compensation by IMRT.

It has been suggested that urinary symptoms following radiation therapy might be related to the maximum dose delivered to the urethra ^(29)^. However, several recent studies indicated that there was no correlation between the maximum urethral dose and urinary symptoms or IPSS ^(30-32^**)**. Bucci *et al*. ^(30)^ reported that all the dosimetric parameters were not correlated with post-implantation catheterization required for patients having obstructive urinary symptoms in a multivariate analysis. They concluded that only the baseline IPSS was the most significant predicative factor for post-implantation catheterization. Crook *et al*. ^(31)^, in a retrospective study, analyzed the results of 150 consecutive patients treated with ^125^I seed implant brachytherapy. Twenty patients (13%) experienced acute urinary retention (AUR). They found that none of the dosimetric parameters, including D_90_, V_100_, V_200_, and maximum urethral dose, was predictive of AUR. In addition, they also found that baseline IPSS did not correlate with AUR after implantation. The prostate volume was the major determinant of AUR. The report by Cesaretti *et al*. ^(32)^ on 172 patients showed that the mean pre-treatment IPSS was 7.5 and the mean peak IPSS post-implantation was 19.4. A total of 35.5% of the patients have experienced a urinary symptom, as determined by the IPSS. Their study found that no single clinical or implant parameters, including PSA, stage, use of hormone therapy, seed activity, prostate volume, seed number, and the urethral dose, had statistically significant correlation with the urinary symptom or the IPSS. In a prospective randomized trial using combined EBRT and seed implant brachytherapy, Merrick ^(33)^ *et al*. found that the isotope type, supplemental EBRT, and the maximum urethral dose did not significantly correlate with the post-treatment IPSS and thus, did not predict for dysuria.

In our present series, our data also showed that no clinical or dosimetric parameters were significantly correlated with the IPSS for both seed implant alone group and the combined modality group. The results agreed with the findings by the investigators mentioned above. Nevertheless, our data showed a weak positive linear correlation between the IPSS and the total maximum urethral dose. In addition, our data also revealed that there was a week linear correlation between the IPSS and the mean urethral dose for the combined modality group. All these suggest that a reduction in mean or maximum urethral dose may result in a decrease in urinary symptoms and the combined modality treatment could induce a higher acute urinary morbidity. This result seemed to be consistent with the popular assumption that combined EBRT and seed implant brachytherapy has higher urinary complications than either therapy alone ^(23)^. It should be pointed out that our post-implant dosimetry was performed three hours after the implant procedure, rather than one month as widely adopted. This was for the sake of patient convenience. We have been aware of the potential impacts of post-implant edema and possible seed migration on the post-implant dosimetry accuracy. Based on the published studies ^(34, 35)^ and our own experience ^(36, 37)^, we believe that our post-implant dosimetry data provided a very conservative estimation of several important dosimetric parameters, such as D_90_, D_100_, and V_100_. This was one of the reasons why our post-implant dosimetry was not favorable as compared with the pre-implant one.

It has been suggested that it could be the dose from above the basal prostate level to the bladder base (bladder neck), rather than the dose to the urethra, that is the stronger predictor of the acute urinary morbidity ^(38)^. This is because this area of the bladder is sensitive to radiation and is often covered by high isodose lines. Thus, it may be beneficial to restrict the dose to this area as much as possible.

**Conclusions**

As a curative therapy, brachytherapy has a long history of being employed in treating localized prostate cancer. Earlier results seemed to be disappointing due to immature technology and inappropriate patient selection. Rapid technological advancements in medical imaging, treatment planning systems, and better understanding of the prognostic factors have revitalized the technique. It is emerging as an indispensable treatment option. Particularly, combined IMRT with seed implant brachytherapy is becoming very appealing to patients who are not willing to undergo radical prostatectomy. It has been shown to be the safest way to deliver a dose higher than 90.0 Gy. However, successful implementation of the technique requires extensive expertise from several different disciplines. It may not be feasible for centers with limited medical physics support. In conclusion, our preliminary study showed that IMRT followed by ^125^I seed implant brachytherapy is a promising and viable treatment technique for patients with high grade and clinically localized prostate cancer. However, it could increase the acute urinary morbidity compared with ^125^I seed implant brachytherapy alone, as assessed by IPSS. In addition, there was no statistically significant correlation between the IPSS and the mean and maximum urethral doses. However, we would like to point out that since the sample sizes of the studied populations were relatively small; it was difficult to accurately determine the effects of urethral dose on the IPSS in a statistical sense and to make a definitive conclusion regarding the treatment outcomes. Thus, further studies using large sample sizes are necessary to validate these preliminary findings. Nevertheless, we hope that the results presented in the current study could be used as a useful reference for other people. We believe that IMRT combined with seed implant brachytherapy presents an alternative opportunity to treat prostate cancer. It utilizes advantages of both modalities. Its full potential needs to be further investigated.

**References**

1. National Cancer Institute. Prostate Cancer Progress Report. 5, (June 2004).

2. Barbiere J, Hanley J, Song Y, Dhaman AP, and Chan MF. Concise Matrix Analysis of Point-Based Prostate Targeting for Intensity Modulated Radiation Therapy, *Technology in Cancer Research and Treatment* **6**(1) 1-10 (2007).

3. Leibel SA, Fuks Z, Zelefsky MJ, Hunt M, Burman CM, Mageras GS, *et al.* Technological advances in external-beam therapy for the treatment of localized prostate cancer. *Semin Oncol*. **30**:596-615 (2003).

4. Eng TY, Thomas CR Jr, Herman TS. Primary radiation therapy for localized prostate cancer. *Urol Oncol*. **7**:239-257 (2002).

5. Zelefsky MJ, Whitmore WF Jr. Long-term results of retropubic permanent 125-iodine implantation of the prostate for clinically localized prostatic cancer. *J Urol*. **159**:23-29 (1997).

6. Ling CC, Burman C, Chui CS, Kutcher GJ, Leibel SA, LoSasso T, *et al.* Conformal radiation treatment of prostate cancer using inversely-planned intensity modulated photon beams produced with dynamic multileaf collimation. *Int J Radiat Oncol Biol Phys*. **35**:721–730 (1996).

7. Burman C, Chui CS, Kutcher GJ, Leibel SA, Zelefsky M, LoSasso T, *et al*. Planning, delivery, and quality assurance of intensity modulated radiotherapy using dynamic multileaf collimator: a strategy for large-scale implementation for the treatment of carcinoma of the prostate. *Int J Radiat Oncol Biol Phys*. **39**:863–873 (1997).

8. Zelefsky M, Fuks Z, Happersett L, Lee HJ, Ling CC, Burman C, *et al*. Clinical experience with intensity modulated radiation therapy (IMRT) in prostate cancer. *Rad Oncol*. **55**:241-249 (2000).

9. Polascik TJ, Pound CR, Deweese TL, and Walsh PC. Comparison of radical prostatectomy and iodine 125 interstitial radiotherapy for the treatment of clinically localized prostate cancer: a 7-year biochemical (PSA) progression analysis. *Urol*. **51**:884-890 (1998).

10. D'Amico AV, Whittington R, Malkowicz SB, Schultz D, Blank K, Broderick GA, *et al.* Biochemical outcome after radical prostatectomy, external beam radiation therapy, or interstitial radiation therapy for clinically localized prostate cancer. *JAMA*. **280**:969-974 (1998).

11. Stokes SH. Comparison of biochemical disease-free survival of patients with localized carcinoma of the prostate undergoing radical prostatectomy, transperineal ultrasound-guided radioactive seed implantation, or definitive external beam irradiation*. Int J Radiat Oncol Biol Phys*. **47**:129–136 (2000)

12. Critz FA, Tarlton RS, Holladay DA. Prostate specific antigen-monitored combination radiotherapy for patients with prostate cancer: ^125^I implant followed by external-beam radiation. *Cancer*. **75**:2383-2391 (1995).

13. Vicini FA, Kestin LI, Stromberg JS, Martinez AA. Brachytherapy boost techniques for locally advanced prostate cancer. *Oncology* (Huntington). **13**:491-499 (1999).

14. Spirou SV, Chui CS. A gradient inverse planning algorithm with dose-volume constraints. *Med Phys*. **25**:321–323 (1998).

15. Chui CS, LoSasso T, Spirou S. Dose calculation for photon beams with intensity-modulation generated by dynamic jaw or multi-leaf collimations. *Med Phys*. **21**:1237–1244 (1994).

16. Lawton CA, Wong M, Pilepick MV, et al. Long-term treatment sequelae following external beam irradiation for adenocarcinoma of the prostate: Analysis of RTOG studies 7506 and 7706. *Int J Radiat Oncol Biol Phys.* **21**:935–936 (1991).

17. Wallner K, Chiu-Tsao ST, Roy J, Arterbery VE, Whitmore W, Jain S, *et al.* An improved method for computerized tomography-planned transperineal 125iodine prostate implants. *J Urol.* 146 (1): 90-95 (1991).

18. Nag S, Beyer D, Friedland J, Grimm P, and Nath R. American Brachytherapy Society (ABS) recommendations for transperineal permanent brachytherapy of prostate cancer. *Int J Radiat Oncol Biol Phys*. 789–799 (1999).

19. Wallner K, Roy J, Zelefsky M, Fuks Z, Harrison L. Fluoroscopic visualization of the prostatic urethra to guide transperineal prostate implantation. *Int J Radiat Oncol Biol Phys*. 29(4): 863-867 (1994).

20. Singh R, Al-Hallaq H, Pelizzari CA, Zagaja GP, Chen A, and Jani AB. Dosimetric quality endpoints for low-dose-rate prostate brachytherapy using biological effective dose (BED) vs. conventional dose. *Med Dosi.* **28**:255-259 (2003).

21. Zelefsky MJ, Fuks Z, Hunt M, Yamada Y, Marion C, Ling CC, *et al*. High-dose intensity modulated radiation therapy for prostate cancer: early toxicity and biochemical outcome in 772 patients. *Int J Radiat Oncol Biol Phys*. **53**:1111–1116 (2002).

22. Mallick S, Azzouzi R, Cormier L, Peiffert D, and Mangin P. Urinary morbidity after ^125^I brachytherapy of the prostate. *BJU* . 555-558 (2003).

23. Peschel RE and Colberg JW. Surgery, brachytherapy, and external-beam radiotherapy for early prostate cancer. *Lancet Oncol*. **4**:233-241 (2003).

24. Potters L. Permanent Prostate brachytherapy: lessons learned, lessons to learn. *Oncology* (Huntington) **14**:981-991 (2000).

25. Chen CT, Waterman FM, Valicenti RK, *et al*. Dosimetric analysis of urinary morbidity following prostate brachytherapy (I-125 vs. Pd-103) combined with external beam radiation therapy. *Int J Cancer*. **96**(suppl):83-88 (2001).

26. Blasko JC, Grimm PD, Sylvester JE, Cavanagh W. The role of external beam radiotherapy with I-125/Pd-103 brachytherapy for prostate carcinoma. *Radiother Oncol*. **57**:273-78 (2000).

27. Critz FA, Williams WH, Levinson AK, *et al*. Simultaneous irradiation for prostate cancer: intermediate results with modern techniques. *J Urol*. **164**:738-43 (2000).

28. Puthawala AA. Syed AM, Austin PA, *et al*. Long–time results of treatment of prostate carcinoma by staging pelvic lymph node dissection and definitive irradiation using low-dose rate temporary irridium-192 interstitial implant and external beam radiotherapy. *Cancer*. **92**:2084-94 (2001).

29. Peschel RE, Chen Z, Roberts K, Nath R. Long-term complications with prostate implants: iodine^125^ vs. palladium, *Radiat Oncol Invest*. **6**:135-141 (1998).

30. Bucci J, Morris WJ, Keyes M, Spadinger I, Sidhu S, and Moravan V. Predictive factors of urinary retention following prostate brachytherapy, *Int J Radiat Oncol Biol Phys*. **53**:91–98 (2002).

31. Crook J, Mclean M, Catton C, Yeung I, Tsihlias J, and Pintilie M. Factors influencing risk of acute urinary retention after TRUS-guided permanent prostate seed implantation. *Int J Radiat Oncol Biol Phys*. **52**:453–460 (2002).

32. Cesaretti JA, Stone NN, and Stock RG. Urinary symptom flare following I-125 prostate brachytherapy, *Int J Radiat Oncol Biol Phys*. **56**:1085–1092 (2003).

33. Merrick GS, Butler WM, Wallner KE, Galbreath RW, Murray B, Zeroski D, *et al*. Dysuria after permanent prostate brachytherapy. *Int J Radiat Oncol Biol Phys*. **55**:979–985 (2003).

34. Yue N, Chen Z, Peschel R, Dicker AP, Waterman FM, Nath R. Optimum timing for image-based dose evaluation of 125I and 103PD prostate seed implants. *Int J Radiat Oncol Biol Phys*. 4**5**:1063-1072 (1999).

35. Chen Z, Yue N, Wang X, Roberts KB, Peschel R, Nath R. Dosimetric effects of edema in permanent prostate seed implants: A rigorous solution. *Int J Radiat Oncol Biol Phys*. **47**:1405-1419 (2000).

36. Willins J, Wallner K. CT-based dosimetry for transperineal I-125 prostate brachytherapy. *Int J Radiat Oncol Biol Phys*. 39 (2): 347-353 (1997).

37. Willins J, Wallner K. Time-dependent changes in CT-based dosimetry of I-125 prostate brachytherapy. *Int J Radiat Oncol Biol Phys*. 6 (4): 157-160 (1998).

38. Williams SG, Millar JL, Duchesne GM, Dally MJ, Royce PL, and Snow RM. Factors predicting for urinary morbidity following ^125^Iodine transperineal prostate brachytherapy. *Radiother Oncol*. **73**:33-38 (2004).

**Figure Legends**

**Figure 1**

Typical isodose distribution for a representative transverse slice of an IMRT plan. The red and green contours represent the PTV and the urethra, respectively. The maximum urethral dose was 100% of the prescribed dose, thus creating a circular low dose region around the urethra.

**Figure 2**

The 95 Gy prescription isodose line (green) for a representative seed implant plan computed for the same patient as shown in **Figure 1.** The pink squares indicate needles used for seed deposition. Three seeds were implanted in this particular slice, indicated by three small white dots.

**Table 1.** Criteria for IMRT plan acceptance

**Physical end points**

**PTV** D_Max_ < 110%

D_Min_ ≥ 87%

D_95_ ≥ 95%

V_95_ ≥ 95%

**Rectal Wall** D_Max_ ≤ 106%

V_79_ ≤ 45%

V_40_ ≤ 90%

**Bladder Wall** D_Mean_ ≤ 60%

**Urethra** D_Max_ ≤ 100%

***Abbreviations*:** PTV = planning target volume

D_Max_ = maximal dose

D_Min_ = minimal dose

D_Mean_ = mean dose

D_95_ = dose covering 90% volume

V_95_, V_79_, V_40_ = volume receiving 95%, 79%, and 40% prescribed dose, respectively

***Note*:** These are the criteria used for 50.4 Gy prostate cases only at MSKCC

**Table 2.** Clinical characteristics of the two study groups

**Parameter IMRT plus seed Seed implant *p*- value**

**implant group (n = 15) alone group (n = 15)**

**Age**

Mean ± SD 63.0 ±7.1 65.7±7.6 0.33

Median 64.0 66.0

Range 49.0~73.0 53.0 ~ 78.0

**Baseline PSA (ng/ml)**

Mean ± SD 7.4 ±4.2 6.0±3.0 0.34

Median 6.92 5.1

Range 3.3~18.8 1.0 ~ 14.0

Prostate Vol (cm^3^)

Mean ± SD 35.4±7.7 33.3±9.7 0.68

Median 35.8 31.7

Range 20.8~54.5 20.3 ~52.2

Stage T1c ~ T2a T1c 0.018

Gleason Score

Mean ± SD 6.9±0.5 6.1±0.4 < 0.001

Median 7 6

Range 6~8 6 ~7

3-Week IPSS

Mean ± SD 12.7±7.4 10.2±7.1 0.38

Median 15.5 7.0

Range 2~26 2~25

4-Month IPSS

Mean ± SD 12.0±8.0 9.5±7.0 0.39

Median 13.5 8.5

Range 2~26 2~ 22

***Abbreviations*:** PSA = prostate specific antigen

IPSS = international prostate symptom score

SD = standard deviation

**Table 3.** Seed implant planning parameters for the two study groups

**Parameter IMRT plus seed Seed implant *p*- value**

**implant group (n = 15) alone group (n = 15)**

**Number of Needles**

Mean ± SD 15.2 ±2.0 18.4±3.2 0.0012

Median 16 18

Range 14~18 14~25

Number of Seeds

Mean ± SD 45.1±8.2 51.6±9.5 0.043

Median 43 52

Range 31~64 44~74

Seeds/Needle

Mean ± SD 3.4±0.8 2.8±0.4 0.33

Median 2.8 2.9

Range 2.1~5.5 2.1~3.4

Activity/Volume (mCi)

Mean ± SD 0.60±0.08 0.86±0.15 < 0.001

Median 0.56 0.85

Range 0.53~0.79 0.47~1.11

Total Activity/Case (mCi)

Mean ± SD 20.93±3.75 27.49±5.5 < 0.001

Median 20.45 26.88

Range 15.62~29.73 20.39~40.79

**Table 4.** Basic pre- and post-implant dosimetric parameters for the

combined modality group (Prescription dose = 95 Gy)

**Dosimetric Pre-implant Post-implant *p*- value**

**Parameter (n = 15) (n = 15)**

**Prostate Vol (cm^3^)**

Mean ± SD 35.4±7.7 32.7±9.8 0.38

Median 35.8 33.4

Range 20.8~54.5 19.3~59.8

D_90_ (Gy)

Mean ± SD 133.7±10.2 112.2±13.4 < 0.001

Median 133.5 110.5

Range 120.9~150.0 93.0 ~ 145.0

D_100_ (Gy)

Mean ± SD 95.9±9.7 81.6±12.2 < 0.001

Median 92.6 75.0

Range 88.4~120.1 75.0~110.0

V_100_ (% prostate vol.)

Mean ± SD 99.0±2.7 94.8±3.9 < 0.001

Median 99.7 95.0

Range 88.4~100.0 88.0~100.0

Max Urethral Dose (Gy)

Mean ± SD 180.6±20.7 168.5±33.4 0.21

Median 174.0 160.0

Range 152.4~227.2 129.0~247.4

**Table 5.** Basic pre- and post-implant dosimetric parameters for the

seed implant alone study group (Prescription dose = 144 Gy)

**Dosimetric Pre-implant Post-implant *p*- value**

**Parameter (n = 15) (n = 15)**

**Prostate Vol (cm^3^)**

Mean ± SD 33.3±9.7 38.2±15.0 0.28

Median 31.7 38.7

Range 20.3~52.2 18.0~62.8

D_90_ (Gy)

Mean ± SD 189.8±9.4 146.9±15.3 < 0.001

Median 191.9 145.0

Range 174.4~205.1 125.0~170.1

D_100_ (Gy)

Mean ± SD 132.1±11.8 109.2±14.5 < 0.001

Median 133.1 109.0

Range 113.0~159.7 85.0~136.8

V_100_ (% prostate vol.)

Mean ± SD 99.7±0.4 92.8±4.7 < 0.001

Median 99.7 93.0

Range 99.4~100.0 82.0~98.0

Max Urethral Dose (Gy)

Mean ± SD 243.8±24.0 224.8±38.5 0.11

Median 248.5 219.0

Range 172.3~279.3 158.9~298.2

**Table 6.** PSA follow-up of the two study groups

**Figure 1**


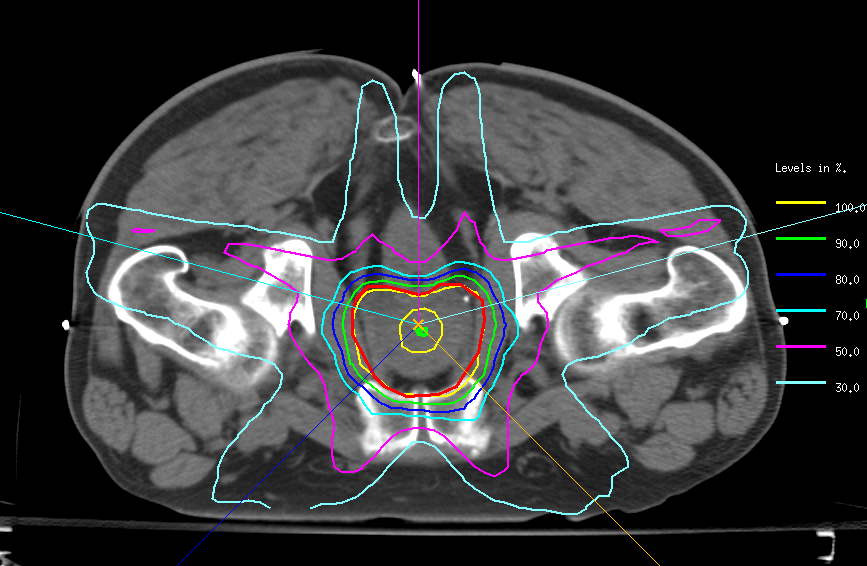


**PTV**

**Urethra**

**Levels in %**

**100.0**

**90.0**

**80.0**

**70.0**

**50.0**

**30.0**

**Figure 2**


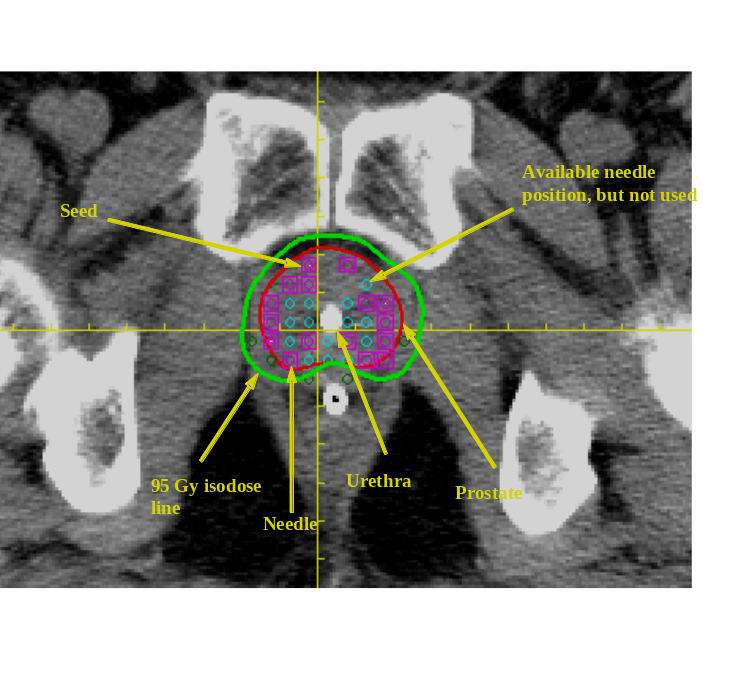

Supplement: Supplementary file 2 — Supplementary Material [file ACM2-9-001-s002.doc]
