# Supplementary material for: Comparison of two treatment approaches for prostate cancer: intensity‐modulated radiation therapy combined with I125 seed‐implant brachytherapy or I125 seed‐implant brachytherapy alone
Source: J Appl Clin Med Phys. 2008 Mar 18;9(2):1–14. doi: 10.1120/jacmp.v9i2.2283 (PMC5721712; doi:10.1120/jacmp.v9i2.2283)
Supplement: Supplementary file 3 — Supplementary Material [file ACM2-9-001-s003.doc]

Comparison of Two Treatment Approaches for

Prostate Cancer: Combined Intensity Modulated Radiation Therapy with ^125^I Seed Implant Brachytherapy and ^125^I Seed Implant Brachytherapy alone

**Yulin Song, Ph.D.^1^*, Maria F. Chan, Ph.D.^1^,**

**Chandra Burman, Ph.D.^1^, and Donald Cann, MD.^2^**

*^1^Department of Medical Physics*

*Memorial Sloan-Kettering Cancer Center at Dover, NJ*

*400 Blackwell Street, NJ 07801*

*^2^Department of Radiation Oncology*

*Memorial Sloan-Kettering Cancer Center at Dover, NJ*

*400 Blackwell Street, NJ 07801*

[songy@mskcc.org](mailto:songy@mskcc.org), [chanm@mskcc.org](mailto:chanm@mskcc.org), [burmanc@mskcc.org](mailto:burmanc@mskcc.org), [dcann@saintclares.org](mailto:dcann@saintclares.org)

Running Title: Combined IMRT with Brachytherapy for Prostate Cancer

***Address Correspondence to:**

Yulin Song, Ph.D.

Department of Medical Physics

Memorial Sloan-Kettering Cancer Center at Phelps

777 N Broadway, Suite 100

Sleepy Hollow, NY 10591

Tel: (914)-333-8676

Fax: (914)-887-8506

E-mail: songy@mskcc.org

Comparison of Two Treatment Approaches for Prostate Cancer: Combined Intensity Modulated Radiation Therapy with ^125^I Seed Implant Brachytherapy and ^125^I Seed Implant Brachytherapy alone

**Abstract:** The purpose of this study was to assess the results of two different treatment approaches for clinically localized prostate cancer: intensity modulated radiation therapy (IMRT) followed by ^125^Iodine (^125^I) seed implant brachytherapy and ^125^I seed implant brachytherapy alone. Thirty most recent consecutive patients were studied. The sample population consisted of 15 cases treated with IMRT (50.4 Gy), followed by ^125^I seed implant boost (95 Gy), and 15 cases treated with ^125^I seed implant only (144 Gy). Established dosimetric indices and various clinical parameters were analyzed. In addition, the acute urinary morbidities of the two treatment approaches, as assessed by the International Prostate Symptom Score (IPSS), were also evaluated and compared. In our series, IMRT followed by ^125^I seed implant brachytherapy slightly increased the acute urinary morbidity compared with ^125^I seed implant brachytherapy alone. In addition, there was no statistically significant correlation between the IPSS and the maximum or mean urethral dose. Combination of IMRT and seed implant brachytherapy presents an alternative opportunity to treat clinically localized prostate cancer. Its full potential needs to be further investigated.

Keywords: Prostate cancer, brachytherapy, IMRT, PSA, and IPSS

PACS: 87.53.Tf

© 2007 American College of Medical Physics
